# Individual and contextual effects of attention in risky choice

**DOI:** 10.1007/s10683-024-09849-7

**Published:** 2024-09-30

**Authors:** Alejandro Hirmas, Jan B. Engelmann, Joël van der Weele

**Affiliations:** Center for Experimental Economics and political Decision Making (CREED), Universiteit van Amsterdam, Amsterdam, The Netherlands

**Keywords:** Attention, Random, Utility models, Eye-tracking, Loss aversion, D81, D83, D87, D91

## Abstract

We investigate the role of visual attention in risky choice in a rich experimental dataset that includes eye-tracking data. We first show that attention is not reducible to individual and contextual variables, which explain only 20% of attentional variation. We then decompose attentional variation into individual average attention and trial-wise deviations of attention to capture different cognitive processes. Individual average attention varies by individual, and can proxy for individual preferences or goals (as in models of “rational inattention” or goal-directed attention). Trial-wise deviations of attention vary within subjects and depend on contextual factors (as in models of “salience” or stimulus-driven attention). We find that both types of attention predict behavior: average individual attention patterns are correlated with individual levels of loss aversion and capture part of this individual heterogeneity. Adding trial-wise deviations of attention further improves model fit. Our results show that a decomposition of attention into individual average attention and trial-wise deviations of attention can capture separable cognitive components of decision making and provides a useful tool for economists and researchers from related fields interested in decision-making and attention.

**Supplementary Information:**

The online version contains supplementary material available at 10.1007/s10683-024-09849-7.

## Introduction

Over the last decades, economists have become increasingly interested in attention. For instance, on the microeconomic level, researchers have proposed that attention may explain behavioral biases such as the endowment effect, the attraction effect or the phenomenon of motivated cognition (e.g., Amasino et al, 2021; Gabaix, 2019). On the macroeconomic level, limits to attention may explain how economic agents react to news shocks, form expectations about future prices and how this affects business cycles (e.g., Sims, 2003). Alongside these applications, several prominent new theories try to incorporate the role of attention in economic behavior. “Salience theory” explains how prominent features among potential payoffs attract attention and sway decisions, leading to behavioral biases (Bordalo et al., 2012, 2013). Theories of “Rational Inattention” propose that decision makers direct limited attentional resources to information that is deemed to be most useful (Gabaix, 2019; Sims, 2010). Finally, sequential sampling models offer a descriptive framework of how processes of information acquisition translate into decision making (Fudenberg et al., 2018; Krajbich et al., 2012; Ratcliff, 1978).

These theoretical approaches differ fundamentally in their description of economic agents. Some theories, like rational inattention, emphasize personal factors such as individual preferences as a source of attention. Others, like salience theory, stress external contextual influences. This discrepancy mirrors a prominent distinction in psychology and neuroscience, where researchers distinguish between “goal-directed” (also referred to as “top-down" or “endogenous”) and “stimulus-driven” (also referred to as “bottom-up” or “exogenous”) attention processes (Corbetta & Shulman, 2002; Egeth & Yantis, 1997; Posner et al., 1980). It is clear from both the neuroscientific literature and everyday experience that both these forces affect attention. As an example, consider going to the supermarket with a prepared shopping list that guides your search, while simultaneously being tempted to make unplanned purchases of highly salient or advertised items.

The distinction between personal and contextual drivers of attention matters for both theoretical modeling and for practical applications. Yet, while some papers in psychology and neuroscience have tried to quantify the influence of these different channels on behavior, as we discuss in more detail below, there has been little work to understand their relative importance. In particular, we aim to identify here how much these attentional variations are predictive of choice, and consequently, how useful they are to the toolkit of empirical economists. These questions become more important as attention measurements such as eye-tracking become cheaper and less challenging to implement. For instance, there have been multiple advances on how to measure attention online, e.g., via mouselabweb (Willemsen & Johnson, 2011), or similar applications in oTree (Hirmas & Engelmann, 2024), and attention can be even measured via internet-connected webcams (Yang & Krajbich, 2021).

In this paper, we propose a novel empirical method to approximate personal and context-driven variation in attention, and illustrate our method in two original experiments on risky choice. Over multiple trials, subjects choose to accept or reject lotteries with equiprobable losses and gains, which vary between trials. While subjects make choices, we record their attention patterns to potential gains and losses using eye-tracking. Our method decomposes attentional variation into two orthogonal dimensions: (1) between-subject variation in attention, measured as the individuals’ average attention to specific attributes, and (2) within-subject variation in attention across trials, which is measured as the deviations from the individual-specific average on each trial. We argue that these measures proxy for personal and contextual drivers in our setting: option attributes vary across trials, but not across decision makers, so between-subject variation in average attention should be mostly associated with personal differences. By contrast, within-subject variation in attention, which keeps personal characteristics of the decision maker constant, proxies for contextual influences.

We first show that attention is not well explained by individual characteristics like age or gender, or contextual elements like the size or screen location of gains and losses. This underlines the additional explanatory power that attention can have as a predictor of choice, which we test next. We find that both between-subject and within-subject variation in attention explain risky choices. Between-subject variations in attention to gains and losses correlates with a measure of individual loss aversion. Using standard random utility models, we show that including average individual variation in attention is a significant predictor of the weight allocated to gains and losses in the decision process. This effect is robustly observed across multiple model specifications and remains significant in the context of statistical methods that capture heterogeneous behavior. In addition, incorporating within-subject attentional variation explains an additional, if modest, amount of variation in choice, suggesting context also has an independent influence on choice. Out-of-sample predictions confirm this trend, showing highly consistent but modest improvements in predictive accuracy across subjects, particularly when average individual attention is additionally modeled.

As we explain in more detail in the next section, we contribute to the literature on attention in economic choice in various ways. First, we show how eye-tracking data can be decomposed into two channels that approximate the individual vs. contextual distinction in attentional control, which is commonplace in the neuroscience and psychology literature (and more recently in economics). We also contribute to the literature on risky choice, by showing that both individual and contextual attentional processes are linked with decisions involving risk. Our results show that risk taking is related to both personal, agent-related characteristics involved in deliberate choices, but also to situational factors such as the salience of specific choice options.

## Related literature

The fields of psychology and cognitive (neuro-)science have long studied attention as a mechanism that reduces demands on limited visual and other cognitive systems by filtering relevant information from the large amounts of information entering our perceptual systems at any moment (e.g. Posner, 2011). Recent key empirical findings that show a strong link between visual attention and decisions have attracted the interest of the field of decision science. Specifically, choice options that enter the attentional focus more often and for longer are more likely to be chosen (Krajbich et al., 2010, 2012; Lim et al., 2011; Pachur et al., 2018; Polonio et al., 2015) and choice options with higher values attract attention more than those with lower values (Anderson et al., 2011; Amasino et al., 2019; Gluth et al., 2018; Gluth et al., 2020).

When it comes to characterizing the determinants of attention, the literature makes a fundamental distinction between goal-directed (top-down) and stimulus-driven (bottom-up) channels of attention, as defined in the introduction. Stimulus-driven attention is thought to have a larger influence on explorative decision processes, when individuals do not yet have a specific rule of choice (Fehr & Rangel, 2011; Gottlieb et al., 2013). Nonetheless, a number of studies have provided evidence that both channels of attention play a role in decision-making (e.g. Corbetta and Shulman, 2002; Orquin and Lagerkvist, 2015; Orquin and Mueller Loose, 2013). Moreover, empirical and theoretical considerations in neuroscience suggest that the brain may process these types of attention in partially separable neural networks (e.g., Corbetta and Shulman, 2002; Kastner and Ungerleider, 2000).

In economic theory, similar distinctions have emerged. The importance of stimulus-driven attention for economic decisions is represented in “salience theory” proposed by Bordalo et al. (2012, 2013) and related models like Kőszegi and Szeidl (2013). These models propose functions that map different choice attributes into “salience”, which reflects the ease by which information is detected by the decision maker. Greater salience of an attribute translates into higher weights of said attribute in the decision. In these models, salience operates in a mechanical way, i.e. without any explicit optimization by the decision maker. It is therefore likely to lead to behavioral biases. Indeed, some of the key insights of these models are to account for a variety of behavioral biases such as the Allais’ paradox or the endowment effect (Bordalo et al., 2012).

By contrast, the importance of goal-directed attention is reflected in economic models of rational inattention (Bartoš et al., 2016; Caplin & Dean, 2015; Gabaix, 2019; Sims, 2003, 2010). In these theories, the decision maker optimally allocates scarce attention to those information sources or attributes that are most likely to affect the utility of choice. These models offer an answer to the question of how a decision maker can optimally allocate attention before actually knowing the value of the choice (Gabaix, 2014). Applications have emerged in finance (Peng & Xiong, 2006), business cycle theory (Maćkowiak & Wiederholt, 2015), monetary policy (Mackowiak & Wiederholt, 2009), industrial organisation (Dessein et al., 2016; Fosgerau et al., 2020), and consumer theory (Caplin & Dean, 2015; Matějka & McKay, 2015; Reis, 2006).

Our exercise is motivated by the seemingly disparate views of the relative roles of agent and context that are inherent in these theoretical approaches. Our goal here is to approximate these attentional processes using readily available measures of attention in laboratory settings, namely eye-tracking, in combination with a novel econometric approach that separates average attention—reflecting individual-differences—from trial-wise deviations in attention—reflecting contextual influences on attention. Most closely related to this endeavor are papers that decompose attention using a number of different methods^1^. Fisher (2021) investigates the role of attention in intertemporal discounting, and shows that both within- and between-subject variation in attention allocation correlate with intertemporal decisions. In addition, random variations in exposure time to different attributes explain about 5% to 10% of intertemporal choices. Ghaffari and Fiedler (2018) attempt to disentangle top-down and bottom-up attentional processes in moral choices. Adapting the well-established empirical result that choices are predicted by the last fixation, they experimentally manipulate the last fixation. Their results indicate that the attribute fixated last is predictive of choice, indicating an effect of bottom-up attention, which they estimate to be responsible for about 11% of the variance in decisions. Third, Towal et al. (2013) perform an eye-tracking experiment on snacks, where they first elicited the value of snacks from participants. They calibrate the parameters of a modified drift-diffusion model (Ratcliff, 1978), where the drift rate can depend on the product’s value and/or salience, a measure constructed from the perceptual features of the products appearance. Value appears as a more important predictor than salience, with a relative weight that is about 3 times higher. Finally, Navalpakkam et al. (2010) ask their participants to choose between multiple targets that vary in value and salience, finding a significant effect of both on the decision.

Our paper adds to this literature by providing a statistical approach that decomposes attentional variation into (1) individual-average attention and (2) deviations from average attention on each trial using the same underlying eye-tracking data. This approach enables the researcher to assess to what extent each attention channel contributes to variance in choices using a single model. We adapt the traditionally used multi-attribute utility models to allow for both individual- and trial-wise variations in attention. In the context of our model, these attention channels can alter the weights for each attribute, thereby affecting decisions. In doing so, we elucidate the assumptions under which one can approximate goal-directed and stimulus-driven processes via between- versus within-subject variation in attention and choice.

Apart from our methodological insights, we contribute to a literature about the role of attention in risky choice (Fiedler & Glöckner, 2012; Pachur et al., 2018). In particular, we complement findings by Pachur et al. (2018), who show that loss aversion parameters are correlated with attention, and that exogenous variations in attention cause shifts in loss aversion. Our paper adds to this evidence, and shows that loss aversion is correlated with between-subject variation in attention. This is in line with our theoretical approach, which associates between-subject variation in attention with mechanisms that are internal to the agent. Additionally, our finding that within-subject variation in attention plays a role in risky choice may help explain the instability of decisions in risky choice across contexts (Bordalo et al., 2012; Johnson & Schkade, 1989).

## Experimental design

### Participants

In total 99 participants took part in two experiments ($n_1 = 53$, $n_2 = 46$), which were identical except for small details (more on that below). Data from 8 participants were excluded because of technical problems that occurred during data collection (5 in Exp.1 and 3 in Exp.2) due to wearing glasses or contact lenses that were incompatible with the eye-tracker (n = 5) and problems with recording the behavioural data (n = 3). One participant made the same decision in all trials, therefore their data was excluded. Partial data for one of two sessions was included for 3 more subjects (2 in Exp.1 and 1 in Exp.2), due to incomplete measurement of the visual data in one of the sessions (data loss of more than 75% due to calibration difficulties). The final data used for analysis therefore contains 91 participants (59 females, average age is 23.5 years). Our participant numbers are comparable to recent eye-tracking studies (e.g., Alós-Ferrer & Ritschel, 2022; Devetag et al, 2016). Moreover, we collected a large number of trials (N=160) per participant, which increases power when within-subject variability across trials is large (Rouder & Haaf, 2018; Shinya & Takiyama, 2024). Given the range of gains and losses offered to participants across trials, such intra-subject variability in choice and attention was expected in the current experiment.

Participants in both experiments were students from the University of Amsterdam, with no impaired or corrected vision. The recruitment was done via the website of the Behavioral Science Lab that houses the eye-trackers used in the current experiment (https://www.lab.uva.nl/lab). The participants signed an informed consent (available in the Appendix) and the experiments were approved by the FMG Ethics Committee of the University of Amsterdam.

### Experimental procedures

On the day of the experiment, participants performed the main task in a darkened testing room. This was done to reduce the effects of ambient light changes on pupil dilation. Jointly, the instructions, practice session and calibration procedures provided ample time to adjust to the background light in the experiment room. Eye movements made throughout the experiment were recorded using an EyeLink 1000 desk-mounted eye-tracker with a sampling rate of 500 Hz. To improve the accuracy of eye-tracking data collection, participants were asked to rest their heads on a chinrest to stabilize the head position and maintain a constant distance from the screen throughout the experiment. The stimuli were presented on a 22-inch screen with the resolution set to 1920 $\times$ 1080 pixels and a refresh rate of 60 Hz. At the start of the experiment and at the half-way point (after 80 trials) a 9-point calibration was performed to ensure proper calibration of the eye-tracker throughout the experiment.

### Main task

**Fig. 1:**
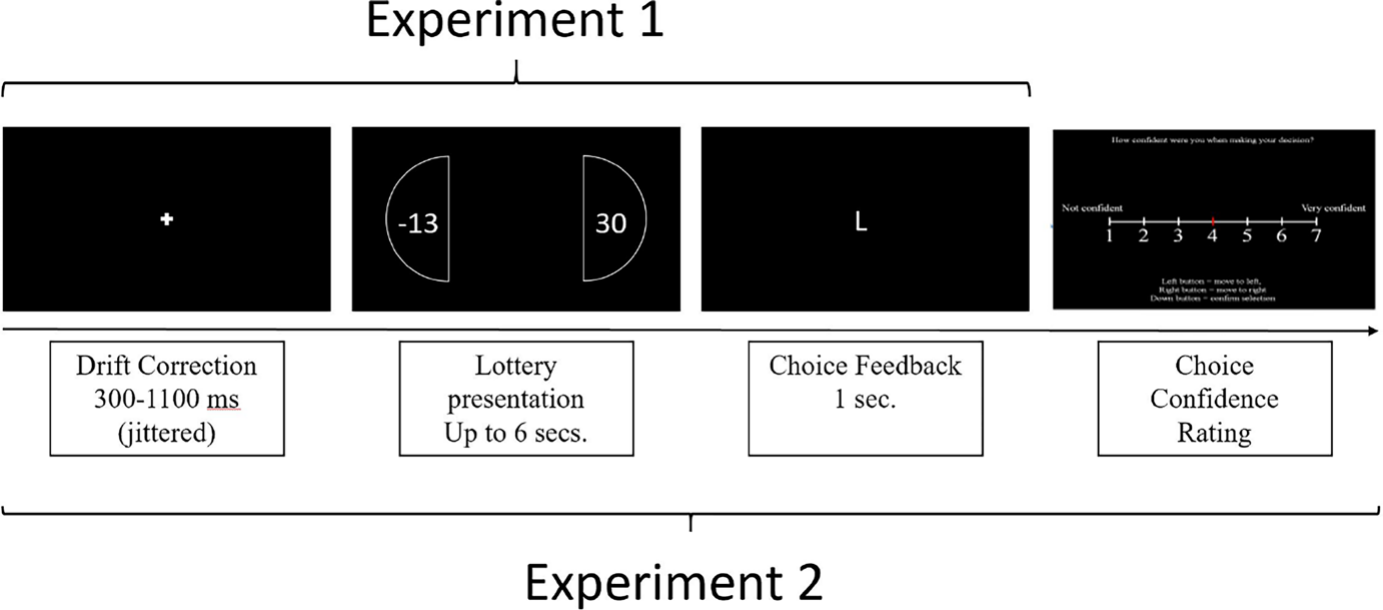
Example of Experimental Trial Initially, a white fixation cross is shown for a random duration that is jittered between 300 and 1100 ms. The prospect is then presented. Participants then communicated their decision by pressing the up or down keys of the keyboard to accept or reject respectively. Feedback informed participants what option they had chosen before the next trial began in experiment 1. Experiment 2 differed only in that participants were asked to rate their confidence before the next trial

The main task in both experiments consisted of a series of 160 individual decisions involving risk. In each trial, participants were asked to accept or reject a mixed gamble with two equally likely outcomes. The outcomes were always a positive (“gains”) and a negative one (“losses”). Figure 1 shows the sequence of an example trial. At the beginning of the trial, participants were asked to focus on a fixation cross presented in the middle of the screen for a jittered period of time (300–1100 ms). This ensured that in each decision period eye fixations started from the same central position and that attention was not biased towards a single location. Then the two potential outcomes appeared at each side of the screen, with the left stimulus located at ($x=480$px, $y=580$px), and the right one at ($x=1420$px, $y=580$px). This wide separation between lottery options along the *x*-dimension (of approximately $25^\circ$ of visual angle) ensured that eye movement patterns can be well separated during the analysis stage (see Figure S1). The location of gains and losses was counterbalanced, such that they had an equal chance of appearing on the left or right in each trial.

The participants were asked to press the Up-Key on the keyboard to accept the gamble or the Down-Key to reject it. Subjects were given a period of 5 s to make the decision. If the subject did not respond within those 5 s, a message appeared on the screen reminding participants to ‘Respond Faster’. Participants were aware that if they did not respond within the 5-second period, they would receive the loss outcome of that trial in case it was selected at random at the end of the experiment. The limit on the decision time was implemented to ensure a predictable and swift experiment for each participant, and to avoid participant fatigue, which can lead to loss of eye-tracking data (e.g., due to closing eye-lids). To provide ample time for decisions while keeping the experiment reasonably short, we estimated the average decision times from prior research using an equivalent task setup (average decision time = 1400 ms; Engelmann and Tamir, 2009; Engelmann et al, 2015; 2017), which matches that of the current study (Mean RT = 1400 ms, SD = 712 ms). To allow for some deviations from the average time during more difficult trials we tripled the average estimate from previous studies and rounded up to 5 s. This approach was successful: In total, only 47 of the 14,372 trials included in the cleaned dataset exceeded the time limit; these ‘miss’ trials were excluded from the analysis. Moreover, in 95.76% of the trials, participants made a decision within 3 s. This means that the time constraint was indeed not binding, and that it was unlikely to impact subjects’ decision process. In experiment 2, the trial continued with a question of how confident the subject was about their decision, which was the only difference between the two experiments (see Fig. 1).

The attributes presented on the left and right were pseudo-randomized, such that the subject would never observe a loss or a gain more than three consecutive times on one side. The values of the Gains and Losses varied across trials. The gains fell between 20 and 38 ECU (experimental currency units) in steps of two units (10 cases). The losses ranged from − 13 to − 27 ECU in steps of two (8 cases). Gains and losses were independent from each other, and participants observed all possible combinations between gains and losses twice (80 trials per session in 2 sessions).

### Incentives and payment

Participants filled out a 30-min online questionnaire consisting of a number of established Personality Questionnaires (e.g., ERQ, STAI, BIS-11) before the main experiment. The participants received €10 as a payment for completing the questionnaires. This amount served as an endowment for the main task to avoid the house money effect (Thaler & Johnson, 1990). Participants were informed that one of the 160 trials would be chosen at random after completion of the experiment. With this approach, each decision in the experiment has an equal chance of being the payout-relevant decision. If on the payout-relevant trial that was selected by chance the participant chose to accept the lottery, then the lottery would be resolved via a virtual coin flip. The outcome would be added to the initial endowment if it was a gain, or subtracted from the initial endowment if it was a loss. If the participant did not accept the lottery, participants were simply paid their initial endowment with no change. The ECUs ranged between − 27 and 38 units and were converted to €  at a rate of $\frac{1}{5.4}$. On average participants earned €10.80 and €10.94 (amounts reflect joint earnings for completing the questionnaire and the experimental trials) in Experiment 1 and 2 respectively.

### Eye-tracking data acquisition and pre-processing

Fixation points were carefully calibrated using a 9-point calibration at two time points in the experiment (before the start of the experiment and after 80/160 completed trials). Furthermore, throughout the experiment, gain and loss attributes were clearly separated by presenting one attribute on the left and another on the right of the center. This clear separation of lottery attributes on the screen allowed us to specify well-defined and non-overlapping regions of interest and thereby to improve the identification of fixations. Next, using k-means, we clustered the fixations along the horizontal axis representing fixation areas for left and right gamble attributes, and central fixation, which occurred only at the beginning of each trial. We ignore the vertical position for clustering, since all the stimuli were positioned at the same vertical location. This allowed us to discriminate between fixations for each outcome (left and right ROI) and central fixations (see Supplementary Fig. G4). Finally, *K*-means clustering was performed for each session separately, as separate calibrations were performed for each session.

Table 1 shows the number fixations for each region of interest by their order of occurrence. A large majority of the first fixations are on the centre (90%), indicating that subjects followed task-instructions to focus on the fixation cross between trials. Most subsequent fixations go to the left first (68.9%), reflecting a commonly observed upper-left location bias (Orquin & Mueller Loose, 2013).

**Table 1:** Number of fixations by order of Fixation and Region of Interest

Fixation	Left	Right	Total
1	10,463	3195	13,658
2	2859	9780	12,639
3	5265	2057	7322
4	922	1906	2828
$>5$	922	876	1798
**Total**	**20,431**	**17,814**	**38,245**

We focus our analyses of the eye-tracking data on the dwell times, defined as the period participants fixate on a lottery attribute throughout one trial. We do this, because dwell times are the dominant measure of attention in the literature. Another measure, the number of saccades or switches of gaze between options, are less informative for our purpose. As shown in Table 1, the majority of trials do not contain more than three fixations, hence this number has little variation across trials and participants.

## Empirical strategy

Our empirical strategy proceeds in several steps. First, we aim to establish that attention is not a direct function of other observable variables like choice attributes or standard demographics, as this would make it unlikely that attention adds explanatory power as a predictor in choice models. Second, we aim to separate attentional variation into individual and contextual drivers to approximate the common distinction in cognitive science between goal-directed and stimulus-driven attention. To accomplish this, we decompose attention into between-subject and within-subject variation as proxies for these drivers. Third, we aim to understand the relation of both types of attentional channels with choice behavior. We model choice via a standard random utility model, which allows us to estimate the decision weights on positive and negative lottery payoffs. Decision weights, in turn, relate to loss aversion. We then correlate the individual average attention with these estimated weights. Finally, we compare how the addition of the two types of attention affects the fit of standard empirical models of risky choice. In the remainder of this section, we discuss these conceptual steps in turn.

Before we proceed it should be noted that, when it comes to attentional measures, eye-tracking provides a rich data set from which different measures of attention can be generated. The most commonly used measures, which are the focus of our analysis, are the number of fixations directed to an area of interest (AoI), the total or relative fixation duration on an area of interest, or which stimulus was attended first/last.^2^ For brevity, we focus on the dwell times (total time spent looking at an attribute, after log transformation) in our main analyses, but add supplementary analyses with alternative measures in the appendix when we cannot include these in a parsimonious manner in the main text.

### Determinants of attention

To contribute to the description of choice behavior, attentional measures should contain information that is not already captured by other (standard) observable variables. Otherwise, variables that correlate highly with attention, and that are collected with greater ease than eye-tracking data, could be used as measures to approximate attentional effects on choice. We therefore identify the correlates of attention by separating individual (between-subjects) and contextual (within-subject) factors and subsequently analyze their contribution to explain variations in attention. Furthermore, we investigate whether the role of individual or contextual factors differs for several attentional measures that can be extracted from eye-tracking data, including variations in the proportion of time subjects fixated on each attribute, the total dwell time,^3^ the number of fixations, what is attended first, and which attribute is attended last.

To this end, we estimate a linear mixed model where we regress attention on observables with random-intercepts for each participant (see appendix A for the regression tables). Based on the estimates of our mixed model, we calculate the best linear unbiased predictors (BLUPs; Liu et al, 2008) for the random intercepts as outlined in detail in Sect. 4.3. BLUPs reflect the individual average attention that is not captured by the other observables included in the regression. We next assess how much of the variance in attention can be explained by each factor using a Shapley value analysis (Lipovetsky, 2021) in which we enter the BLUPs as an additional individual factor. The Shapley value regression analysis is a method used to determine the contribution of each covariate in a regression model to the prediction of the target variable,^4^ This analysis not only provides an easier interpretability of the results compared to a standard regression approach, it also provides additional insights when we expect covariates to be highly correlated, and when standard regression approaches are less reliable.

### Attention decomposition

As we argued in the introduction, attention may affect choice via both individual and contextual variation. To understand this better, we decompose attentional variation into two orthogonal measures. First, we calculate each individual’s average attention for each specific stimulus/attribute (i.e., Gains and Losses). Let $a_{x,i,t}$ be the allocated attention to attribute $x\in \{Gain,Loss\}$ by individual *i* in trial $t\in \{1,...,T\}$. We define the average-attention measure ${\bar{a}}_{x,i}$ as the standardized average attention to attribute *x* by individual *i* across all trials.

1 $$\begin{aligned} {\bar{a}}_{x,i}:= & \frac{\frac{1}{T}\left( \sum \limits _{t=1}^Ta_{x,i,t}\right) -\bar{a}_x}{sd(a_x)}. \end{aligned}$$

where ${\bar{a}}_x$ and $sd(a_x)$ are the sample mean and standard deviation of the attention measure for attribute *x*. Thus, this variable reflects *how much more participant* *i* *attends to attribute* *x* *compared to the whole sample* (measured in standard deviations).

Our second measure of attention captures the trial-wise deviations of attention from the individual averages for each attribute. The trial-wise deviation of attention, defined as ${\tilde{a}}_{x,i,t}$, is calculated as in Eq. (2). This variable reflects *how much more participant i attends to attribute x in trial t compared to the participants’ average behaviour* (measured in standard deviations).

2 $$\begin{aligned} {\tilde{a}}_{x,i,t}:= & \frac{a_{x,i,t} - \frac{1}{T}\sum \limits _{t=1}^Ta_{x,i,t}}{sd(a_x)} . \end{aligned}$$

In our study, participants do not receive any feedback about their decisions until the end of the experiment. The only variables that vary across the trials are the values of the lottery outcomes and where they are presented. All participants undergo the same trials but in a different order. This implies that between-subject variation in attention reflects personal differences, and can thus be considered a proxy for attention driven by personal goals and characteristics, which is often associated with goal-directed or top-down attention. By contrast, within-subject variation in attention keeps individual characteristics constant, and hence reflects elements of the decision context, such as the location of gains and losses on the screen (which was randomized). Note that this interpretation of our decomposition relies on some untested assumptions, most notably the absence of interactions between contextual variables and the goals or preferences of individuals. An example for the presence of such interactions is when participants differentially attend to an attribute once the attribute value crosses a threshold (e.g., losses become more important if they have a value that is higher than some reference point). In such a case, this individual effect will be partially captured by trial-wise deviations of attention instead of the average attention. If such interactions exist, the model will overestimate the importance of contextual factors, as individual variations will be wrongfully attributed to context.

### Modeling the decision process

The key part of our analysis concerns the role of attention in decision making. To conceptualize the decision making progress, we use a standard random utility model (RUM; McFadden, 1980). The general specification of our model is described by Eqs. (3) and (4).

3 $$\begin{aligned} P(D_{i,t}=accept)= & (1+\exp \{V_{i,t}\})^{-1} , \end{aligned}$$

4 $$\begin{aligned} V_{i,t}= & \alpha _i + \omega _{G,i}G_{t} + \omega _{L,i}L_{t}. \end{aligned}$$

Here, $\omega _{G,i}$, and $\omega _{L,i}$ can be interpreted as weights on the potential gains and losses that determine the value of the lottery and hence the probability to accept it.

We estimate the RUM using a logit mixed model with random intercepts and slopes. We allow the covariance matrix of the random intercepts and slopes to be unstructured (i.e., non-zero covariance between errors). Additionally, we estimate the best linear unbiased predictors (BLUPs; Liu et al, 2008) for $\alpha _i,\omega _{G,i}$, and $\omega _{L,i}$ for each individual. Mixed models assume that each of the parameters is composed of a mean and an individual error term (i.e., random slopes and intercepts), which are not directly estimated but included in the covariance matrix of the decision models. Therefore, mixed models estimate the distribution of these individual errors. Based on these estimated distributions and the individual decision data, we can calculate the posterior expectation of these errors (i.e., BLUPs). BLUPs thus reflect predictions for the individual parameters that come from a common distribution, but also incorporate the components of the individual decisions.

The goal of our main analyses is to test whether the attention indices can capture the differences in the individual model parameters ($\alpha _i, \omega _{G,i}$ and $\omega _{L,i}$). There are two components to this goal: first, on the behavioral level we wish to confirm that attention correlates with these individual parameters, which would indicate that attention is an important cognitive process that supports choice; second, on the modeling level we wish to demonstrate that we can credibly use attention as a proxy for the individual parameters reflecting heterogeneous behavior in contexts where these parameters are unidentifiable. This can occur for instance in the context of mixed regression models, which can increase in complexity to the point of becoming unidentifiable, especially when aiming to fulfill the common requirement of using maximal random effects structures (Barr et al., 2013). We aim to test whether the use of attention variables offers a parsimonious solution for cases where the model complexity is high and these models are not identifiable.

To address the first goal, we correlate the individual parameters of choice with individual attention variables. We then ask how much of the variability in the individual parameters can be explained by individual average attention and other individual characteristics. To do so, we use a Shapley value analysis described above. The Shapley value regression analysis provides a fair and consistent estimation quantifying the contribution of each attention variable despite the potentially high degree of correlation between them. The Shapley-value analysis is performed using the SHAPLEY2 package in Stata17 (Wendelspiess Chávez Juárez, 2015).

### Effects of separate attention channels on choice

Finally, we analyse whether the different measures of attention can improve empirical models of choice. To accomplish this, we estimate the model defined in Eqs. (3) and (4) and incorporate the attentional measures as moderators for the weights on gain and loss values, as follows:

5 $$\begin{aligned} V_{i,t}= & \alpha _i + \omega _{G,i}G_{t} + \pi _{G,{\bar{a}}}{\bar{a}}_{G,i} + \pi _{G,{\tilde{a}}}{\tilde{a}}_{G,i,t} + \omega _{L,i}L_{t} + \pi _{L,{\bar{a}}}{\bar{a}}_{L,i} + \pi _{L,{\tilde{a}}}{\tilde{a}}_{L,i,t} + \epsilon _{i,t} \end{aligned}$$

where the main effects of the gains and losses are represented by $\omega _{G,i}$ and $\omega _{L,i}$ respectively. The moderating effects of individual-average attention are captured by $\pi _{G,{\bar{a}}}$ for the gain values and $\pi _{L,{\bar{a}}}$ for the loss values. Similarly, the parameters $\pi _{G,{\tilde{a}}}$ and $\pi _{L,{\tilde{a}}}$ capture the moderating effects of the trial-wise deviations of attention to gain and loss values respectively.

To evaluate the contribution of attention to explaining decisions, we consider two types of often-used logit models. First, we consider models that have random intercepts but a common slope, i.e. $\omega _{i}=\omega$, which is a standard approach in economics. To evaluate the role of attention, we estimate the model with and without interactions of individual attention and trial-wise attention to the attributes (gains and losses), and compare standard measures of model fit.

Second, we address our second main goal outlined in Sect. 4.3 and consider more elaborate mixed models (Barr et al., 2013) that include random effects for the coefficients of the attributes (slopes). These models benefit from a large number of observations per person that are typically elicited in neuroscientific experiments, but not economic ones. We can exploit the large dataset we have collected (up to 160 observations per participant), which enables us to estimate these types of models. Importantly, this approach allows us to test the contribution of attention to the model fit after including the individual heterogeneity.

## Results

We now discuss the results of our three-part empirical analyses in turn. Specifically, we first assess the determinants of attention by testing whether variance in attention is associated with individual characteristics like age or gender, or contextual elements like the size or screen location of gains and losses. We find that among the variables included in the current study both individual and contextual factors explain relatively little in the variance of attention, suggesting that including attention as a predictor of choice can add important explanatory power above and beyond these factors. Next, we test exactly this question: does attention explain decisions? Moreover, we compare the descriptive power of three different measures of attention that can be derived from eye-tracking data, a novel decomposition of the eye-tracking data into two attention channels reflecting (1) individual average attention, (2) trial-wise deviation from average attention, and (3) a measure that does not separate attentional variation (the standard in prior research). To this end we sequentially add the different attention variables as predictors into standard random utility models. Specifically, we assess whether average individual variation in attention and trial-wise deviations in attention are significant predictors of choice by testing their effects on decision weights for gains and losses. We do this first for a type of model commonly used in economics that includes random effects for the intercepts. We then compare our results to a model class more commonly used in psychology/neuroscience, namely mixed models that add random slopes for these weights.

### Determinants of attention

In the first part of our analysis, we explore potential factors that influence attention. Tables 2 and 3 show the Shapley value analysis for all the variables measuring attention to Gain and Loss values respectively. In Appendix section A we present the estimates of the regressions used in this analysis.

**Table 2:** Shapley value analysis assessing the explained variance for individual differences in attention to gains

	Prop. DT	DT	N	First	Last
**Contextual factors**	**4.58**	**3.92**	**2.54**	**26.95**	**1.62**
Trial	0.09	0.67	0.56	0.01	0.06
$\text {Trial}^2$	0.01	0.10	0.05	0.00	0.00
Gain value	0.09	0.28	0.19	0.01	0.03
Loss value	0.52	0.23	0.34	0.22	0.40
Loss left	3.87	2.64	1.40	26.71	1.13
**Individual factors**	**3.45**	**20.44**	**16.61**	**0.18**	**3.08**
Female	0.00	0.53	0.02	0.00	0.00
Age	0.17	0.66	0.73	0.00	0.32
Random effects	3.28	19.25	15.86	0.18	2.76
**Total % variance explained**	**8.04**	**24.37**	**19.14**	**27.13**	**4.69**

The table above shows the Shapley value analysis (based on $R^2$) for the attention to gains. The table shows the contribution of each variable (in percentage points) to the variance of each dependent variable. The last row shows the total contribution of all factors to explaining the variance of the attention variables (measured using the $R^2$)

We separate the measured factors as contextual (within subjects) and individual (between subjects) effects. Tables 2 and 3 show that the individual factors explain about 20% and 16% (depending on whether the estimates are for gains or losses) of the total dwell times and the amount of fixations, respectively. Similarly, our results show that the first fixation is strongly explained by the position of the attribute. In all, the contextual and individual factors included here explain a relatively small proportion (between 5% and 25%) of attentional variation, and explain less than 10% of the variable most commonly used as a proxy for attention, namely proportional dwell time.

**Table 3:** Shapley value analysis assessing the explained variance for individual differences in attention to losses

	Prop. DT	DT	N	First	Last
**Contextual factors**	**4.58**	**1.29**	**3.30**	**26.95**	**1.62**
Trial	0.09	0.18	0.31	0.01	0.06
$\hbox {Trial}^2$	0.01	0.03	0.04	0.00	0.00
Gain value	0.09	0.02	0.06	0.01	0.03
Loss value	0.52	0.18	0.13	0.22	0.40
Loss left	3.87	0.88	2.76	26.71	1.13
**Individual factors**	**3.45**	**19.98**	**15.79**	**0.18**	**3.08**
Female	0.00	0.45	0.02	0.00	0.00
Age	0.17	0.09	0.31	0.00	0.32
Random effects	3.28	19.44	15.46	0.18	2.76
**Total % variance explained** ($\varvec{R^2}$)	**8.04**	**21.27**	**19.09**	**27.13**	**4.69**

The table above shows the Shapley value analysis (based on $R^2$) for the attention to losses. The table shows the contribution of each variable (in percentage points) to the variance of each dependent variable. The last row shows the total contribution of all factors to explaining the variance of the attention variables (measured using the $R^2$)

### Decisions and individual differences

Now that we have established that attention shows little association with a number of individual and contextual factors in the previous section, we aim to address our main research question in the current section, which is whether attention is related to decisions. Before estimating decision models with attention variables in the next section, we first assess simple associations between attention and loss aversion. We follow prior work (Pachur et al., 2018) and test the relation between loss aversion and the individual attention measures. To this end, we compute two variables: (1) the difference in the decision weights extracted from a decision model without attention (See Table 6, column 1) and (2) the difference in individual average attention between gains and losses for our three main attention variables (proportional Dwell Time (DT), log(DT), number of fixations). Note that we estimate decision weights using Eq. (4) and a logit mixed model with random intercepts and slopes. This approach enables us to subsequently derive the best linear unbiased predictions (BLUPs) of the individual model parameters, including the intercept ($\alpha _i$), and the individual decision weights for gains ($\omega _{G,i}$) and losses ($\omega _{L,i}$). We then correlate the difference between the estimated individual weights on gains and losses $\Delta \omega =\omega _{L,i}-\omega _{G,i}$, a proxy for loss aversion, with a measure reflecting "attentional" loss aversion, namely $\Delta {\bar{a}} = {\bar{a}}_{L,i} - {\bar{a}}_{G,i}$.

**Fig. 2:**
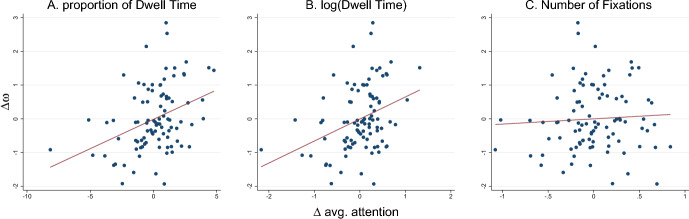
Association between attentional and behavioral loss aversion. Correlation between the differences in decision weights ($\Delta \omega =\omega _{L,i}-\omega _{G,i}$, on the vertical axis) reflecting behavioral loss aversion, and the differences in average attention towards losses relative to gains ($\Delta {\bar{a}} = {\bar{a}}_{L,i} - {\bar{a}}_{G,i}$, on the horizontal axis), reflecting attentional loss aversion. The red line displays the linear fit between the differences in weights and the differences in attention. The differences in attention are standardized

Figure 2 shows the results, with the three panels corresponding to proportional dwell time (Panel a), the log of dwell time (Panel b) and the number of fixations (Panel c). The first two relative attention variables capture a sizeable part of the variation in loss aversion: the proportion of time fixated on losses vs gains ($\rho = 0.3679,p<0.001$) and the total dwell times on losses relative to gains ($\rho = 0.3532,p=0.0005$) are significantly correlated with the differences in decision weights. Note that these results are also significant when applying the Bonferroni correction for multiple comparisons (for the 6 correlations inspected here, the corrected alpha level equals 0.0083). By contrast, the differences in number of fixations have no significant correlation with the individual differences in decision weights ($\rho = 0.0572,p=0.5859$). This shows that individual average attention is indeed a predictor of individual heterogeneity in choice, and individual loss aversion. In Appendix section B, we show a similar analysis for the base level, reflected by the individual intercepts ($\alpha _i$), which represents the individual pre-disposition to accept the lottery regardless of the outcomes. We show that while such predispositions still correlate with attentional measures, these effects are very small.

To get a better sense of the relative predictive power of different attention measures, we run a Shapley value analysis to determine how much of the differences in individual parameters can be explained by the attention variables. Table 4 shows the Shapley value analysis for the individual differences in the decision parameters based on individual characteristics and attentional variables. The first two columns represent the percentage of variance explained for the weights of Gain ($\omega _{G,i}$) and Loss ($\omega _{L,i}$) values respectively. The third column shows the percentage of variance explained for the difference between these decision weights ($\omega _{G,i}-\omega _{L,i}$). Finally, column four shows the explained variance of the intercept ($\alpha _i$). We use as explanatory variables the attentional variables including proportion of time, total dwelling times, number of fixations, first and last fixation, as well as individual characteristics (gender and age).

**Table 4:** Shapley value analysis assessing the explained variance for individual differences in decision parameters

	$\omega _{G,i}$	$\omega _{L,i}$	$\Delta \omega$	$\alpha _i$
**Attention**	**21.96**	**7.13**	**33.21**	**23.16**
prop. (DT)	4.51	1.93	11.54	7.37
ln(DT)	5.83	2.22	7.45	6.61
N	7.87	1.15	6.87	3.79
Last	2.79	0.58	3.36	2.05
First	0.96	1.25	3.99	3.34
**Ind. characteristics**	**8.92**	**6.47**	**6.76**	**7.05**
Female	5.14	6.09	0.11	1.08
Age	3.78	0.38	6.65	5.97
**Total % variance explained** ($R^2$)	**30.88**	**13.6**	**39.97**	**30.22**

The table above shows the Shapley value analysis (based on $R^2$) for the individual attributes $\omega _{G,i}$, $\omega _{L,i}$, their difference $\Delta \omega$ and the intercept $\alpha {i}$. The table shows the contribution of each variable (in percentage points) to the variance of each dependent variable. The last row shows the total contribution of all factors to explaining the variance of the decision parameters (measured using the $R^2$)

The results show that, after controlling for the individual characteristics, a large percentage (up to one third for the difference between weights for losses and gains) of the heterogeneity in decision processes is explained by the individual differences in attention. Predominantly, we find that the proportion of time, total dwelling times and number of fixations contribute most to explaining these differences.

### Incorporating attention in empirical decision models

While the previous results provide evidence for the importance of attention in choice, in this section we aim to establish a closer connection between choice and attention by allowing different attention variables to directly impact the decision weights associated with each attribute in our model. We furthermore ask whether the inclusion of attention variables can improve the estimates of empirical decision models and enhance model fit. Using this approach we compare the explanatory power of the different attention indexes with each other. To this end, we explicitly introduce our attention measures into our estimation of a structural decision model, as illustrated in Eq. 4. For the analysis in this section, we use the log-dwell times as our attention variable, since this measure provides the best fit, based on the Bayesian Information Criterion (BIC). In Appendix section C, we present the same analysis with alternative attention measures.

We first consider a model with random effects for the intercepts, as is common practice for estimating random utility models in economics. Table 5 shows the result of this estimation. For comparison, model 1 includes no measure of attention, and model 2 includes a single standard measure of attention that is commonly used in the literature. Following the notation from Sect. 4, the variable *a*(*x*) represents the attention allocated to attribute *x*. We next decompose attention into two separate channels with ${\bar{a}}(x)$ representing the individual-average attention, and ${\tilde{a}}(x)$, reflecting the trial-wise deviations of attention. These separate attention measures are sequentially introduced in models (3–5), with model 5 reflecting the full model that includes both attention channels. More specifically, we add overall attention to gains and losses (model 2), average individual attention to gains and losses (model 3), trial-wise deviations in attention to gains and losses (model 4) and the combination of individual attention and trial-wise attention (model 5). In each case, we include the interaction with the actual attributes to capture attentional moderation of the decision weights.

Results from the standard model (without attention, model 1) provide evidence for loss aversion, as weights on losses are higher than those on gains. Importantly, for the attention measures we observe that the coefficients for the interaction between individual attention and loss and gain values are statistically significant (model 3), indicating that individual differences in attention capture part of individual heterogeneity in the response to gain and loss size. The direction of the results reflects that spending more attention on gain values leads to an increased sensitivity towards gain amounts. More specifically, participants that spend more attention to gains are more likely to accept lotteries that have higher gain values, and less likely to reject lotteries with low gain values (relative to participants that spend less time focusing on gains). Similarly, increased attention to losses is associated with increased sensitivity to loss amounts. Note that Loss is encoded as signed negative values, thus a positive weight expresses a negative relationship between loss value and the probability to accept the lottery. These findings corroborate the evidence on the relationship between attention and loss aversion reported in the previous section. Turning to trial-wise attention, we find a significant moderating impact on the decisions. Specifically, in the ATT(TW) model (model 4, Table 5) that contains as its only attention parameter the trial-wise deviations of attention, we find that trial-wise attention significantly moderates the gain values but not the loss values, while in the ATT(ID+TW) model (model 5, Table 5), which contains both attention parameters, the loss, but not the gain values are significantly moderated by trial-wise deviations of attention. This means that the significance of the trial-wise attention parameters from the ATT(TW) changes upon the addition of the average attention parameters in the ATT(ID+TW) model. When comparing Table 5 (random intercept models) and 6 (random intercept and random slopes models), we find that the ATT(ID), ATT(TW) and ATT(ID+TW) models (models 3–5) in Table 6 parallel the results from the ATT(ID) and ATT(ID+TW) models (models 3 and 5) in Table 5. Overall, this indicates that models including individual average attention parameters produce stable results across different model specifications, but results related to trial-wise deviations in attention depend on exact model specification. Note that this finding is likely specific for our experimental setting and that experimental designs that manipulate saliency and include more than two attributes can induce more trial-wise variation in attention, and hence yield more stable trial-wise attention parameters.

We next evaluate how the inclusion of attention affects the explanatory power of these models. The measures of model fit at the bottom of Table 5 show that attention-based models have higher explanatory power than the basic model in column 1, as evidenced by the AIC and BIC criteria ($\Delta$ BIC = 190.4 when comparing the baseline model without attention with the two-channel attention model, Att(ID+TW)). Moreover, splitting attentional variation into its individual and contextual components leads to a better fit than including attention without any decomposition (the standard measure in the literature; $\Delta$ BIC = 127.4). The best model fit is reached by including both average and trial-wise attention (AIC/BIC, $\Delta$ BIC with next-best model Att(ID) = 32.4). This shows that including attention leads to more accurate predictions, in general, and that the inclusion of separate attention channels reflecting individual and contextual components leads to further improvements.

**Table 5:** Assessment of the relevance of separate attention channels across decision models estimated with random intercepts

	(1)	(2)	(3)	(4)	(5)
	Baseline	Attention	Att(ID)	Att(TW)	Att(ID+TW)
Gain	0.352$$^{***}$$	0.352$$^{***}$$	0.360$$^{***}$$	0.355$$^{***}$$	0.361$$^{***}$$
	(0.000)	(0.000)	(0.000)	(0.000)	(0.000)
Gain $\times$ *a*(*G*)		0.018$^{*}$			
		(0.030)			
Gain $$\times$$ $${\bar{a}}(G)$$			0.076$$^{***}$$		0.074$$^{***}$$
			(0.000)		(0.000)
Gain $$\times$$ $${\tilde{a}}(G)$$				−0.027$$^{**}$$	−0.006
				(0.004)	(0.445)
Loss	0.433$$^{***}$$	0.430$$^{***}$$	0.446$$^{***}$$	0.434$$^{***}$$	0.443$$^{***}$$
	(0.000)	(0.000)	(0.000)	(0.000)	(0.000)
Loss $$\times$$ *a*(*L*)		0.043$$^{***}$$			
		(0.000)			
Loss $$\times$$ $${\bar{a}}(L)$$			0.077$$^{**}$$		0.095$$^{***}$$
			(0.004)		(0.000)
Loss $$\times$$ $${\tilde{a}}(L)$$				0.008	0.034$$^{***}$$
				(0.647)	(0.000)
*a*(*G*)		−0.370			
		(0.118)			
${\bar{a}}(G)$			−0.677		−0.528
			(0.262)		(0.398)
$${\tilde{a}}(G)$$				0.993$$^{***}$$	0.338
				(0.001)	(0.131)
*a*(*L*)		0.691$$^{**}$$			
		(0.003)			
${\bar{a}}(L)$			−0.148		0.118
			(0.836)		(0.868)
$${\tilde{a}}(L)$$				0.008	0.520$$^{**}$$
				(0.980)	(0.003)
Constant	−1.986$$^{***}$$	−2.058$$^{***}$$	−2.034$$^{***}$$	−2.062$$^{***}$$	−2.139$$^{***}$$
	(0.000)	(0.000)	(0.000)	(0.000)	(0.000)
Observations	14238	14238	14238	14238	14238
*AIC*	8823.290	8730.009	8635.031	8752.162	8572.371
*BIC*	8868.672	8805.646	8710.668	8827.798	8678.262

*p*-values in parentheses

$$^{*}$$ $$p<0.05$$, $$^{**}$$ $$p<0.01$$, $$^{***}$$ $$p<0.001$$

This table shows the estimations of a logit regression with random intercepts. We use log(dwell-times) as our attention measure for this regression. Column (1) shows a baseline model with no attention variables, column (2) includes the standard attention variable (log(dwell-times)) without any decomposition. Next, we include our attention indexes separately (individual average attention in column (3) and trial-wise deviations in column (4). Finally, column (5) includes both attention indexes simultaneously

Next, we compare these results to mixed models that explicitly incorporate individual heterogeneity and are often used in the neuro-economic and cognitive psychology literature. To this end, we estimate the same decision model from Eq. 4, but include individual random slopes for the decision weights ($\omega _{G,i}$ and $\omega _{L,i}$). We expect attentional variables, in particular individual average attention, to have a lower impact relative to the model with exclusively random intercepts, as behavioral heterogeneity is also captured by  the random slopes.

Table 6 shows the estimations of the decision model, where the columns follow the specifications in Table 5: The first column displays the baseline model used for the analysis (i.e., mixed-model with random slopes and intercepts; and no attention variables). The second column incorporates the attention variable without decomposition into separate channels. Columns three and four incorporate the individual-differences and the trial-wise deviations of attention separately, while the last column incorporates both of them simultaneously.

**Table 6:** Assessment of the relevance of separate attention channels across decision models estimated with random slopes and intercepts

	(1)	(2)	(3)	(4)	(5)
	Baseline	Attention	Att(ID)	Att(TW)	Att(ID+TW)
Gain	0.440$$^{***}$$	0.440$$^{***}$$	0.440$$^{***}$$	0.442$$^{***}$$	0.441$$^{***}$$
	(0.000)	(0.000)	(0.000)	(0.000)	(0.000)
Gain $\times$ *a*(*G*)		0.003			
		(0.732)			
Gain $$\times$$ $${\bar{a}}(G)$$			0.061$$^{***}$$		0.060$$^{**}$$
			(0.000)		(0.001)
Gain $\times$ ${\tilde{a}}(G)$				−0.006	−0.002
				(0.470)	(0.789)
Loss	0.582$$^{***}$$	0.576$$^{***}$$	0.581$$^{***}$$	0.577$$^{***}$$	0.575$$^{***}$$
	(0.000)	(0.000)	(0.000)	(0.000)	(0.000)
Loss $$\times$$ *a*(*L*)		0.035$$^{***}$$			
		(0.000)			
Loss $$\times$$ $${\bar{a}}(L)$$			0.050$$^{*}$$		0.070$$^{**}$$
			(0.048)		(0.005)
Loss $$\times$$ $${\tilde{a}}(L)$$				0.036$$^{***}$$	0.037$$^{***}$$
				(0.000)	(0.000)
*a*(*G*)		0.075			
		(0.724)			
${\bar{a}}(G)$			−0.323		−0.199
			(0.610)		(0.760)
${\tilde{a}}(G)$				0.331	0.218
				(0.155)	(0.337)
*a*(*L*)		0.531$$^{**}$$			
		(0.004)			
${\bar{a}}(L)$			−0.745		−0.458
			(0.282)		(0.503)
$${\tilde{a}}(L)$$				0.560$$^{**}$$	0.568$$^{**}$$
				(0.003)	(0.002)
Constant	−1.588$$^{*}$$	−1.710$$^{*}$$	−1.812$$^{**}$$	−1.793$$^{**}$$	−1.971$$^{**}$$
	(0.011)	(0.017)	(0.005)	(0.009)	(0.002)
Observations	14238	14238	14238	14238	14238
*AIC*	8016.990	7967.962	8003.691	7966.473	7953.576
*BIC*	8100.190	8081.417	8117.146	8079.928	8097.286

*p*-values in parentheses

$$^{*}$$ $$p<0.05$$, $$^{**}$$ $$p<0.01$$, $$^{***}$$ $$p<0.001$$

This table shows the estimations of a logit regression with random intercepts and slopes. We use log(dwell-times) as our attention measure for this regression. Column (1) shows a baseline model with no attention variables, column (2) includes the standard attention variable (log(dwell-times)) without any decomposition. Next, we include our attention indexes separately (individual average attention in column (3) and trial-wise deviations in column (4). Finally, column (5) includes both attention indexes simultaneously

We highlight a number of results. First, as in the previous model, measures of individual-average attention are significant moderators for both Gains and Losses in the models of column (3) and (5). Thus, attention patterns continue to reveal additional information about individual heterogeneity, and do so beyond what is captured by the random effects. Moreover, models (4) and (5) show a significant direct (main) effect of the trial-wise deviations of attention to losses, as well as an interaction with loss size. This shows that both individual attentional indexes and salience have an independent influence on the decision process.

Turning to model fit, based on the Bayesian Information Criteria (BIC), the model using the trial-wise deviations of attention is the best fitting model (although the full model still shows a better fit than the model without attention, $\Delta$ BIC = 2.9, and is the best model based on and AIC). Thus, trial-wise attention can improve the model fit beyond individual heterogeneity captured by the random slopes. However, compared to the large improvements in model fit observed after including attention in random intercept models commonly used in economics (delta BIC = 190.4), while the gains of including attention remain large for models containing both random intecepts and slopes ($\Delta$ BIC = 20.3), they are relatively smaller. This is especially true when compared to the large gain in fit obtained from incorporating random effects for the loss and gain size variables ($\Delta$ BIC between baseline models = 758.5; compare column 1 in Tables 5 and 6).

Overall, we conclude that attentional variables emerge as significant correlates of choice and capture individual heterogeneity, even in models that include random slopes. Moreover, they lead to large improvements of model fits in standard economic models with common slopes, especially when decomposing attention into two channels, namely individual average attention and trial-wise deviations of attention. Finally, our results show relatively smaller gains in model fits for mixed models that include both random intercepts and random slopes, but adding separate attentional channels still leads to superior model fits also in this class of models.

## Discussion and conclusion

In this study, we investigate the relationship between attention and the decision process. Participants take part in an incentivized experiment involving lotteries with positive and negative outcomes, while their eye movements are recorded using eye-tracking. We analyze: (1) the factors influencing attention, (2) how attention patterns relate to individual differences in decision-making, (3) whether including attention in commonly used models for decision-making processes can enhance model fit, and (4) whether a single attention measure or a decomposition into two attention channels reflecting individual average attention and trial-wise deviations of attention further improves model fit.

Our results show that attention is weakly associated with both contextual factors, such as the position of the information, and individual factors, such as gender and age. Importantly, while some factors are significantly correlated with variations in attention, their contribution is small, leaving most of the variance in attention unexplained. These results suggest that measures of attention share little variance with common measures related to individual and contextual factors, and are therefore in a position to explain components of the variance of decision making processes above and beyond these measures.

This is exactly what our subsequent results show. Specifically, we first demonstrate significant correlations between decisional and attentional loss aversion. We subsequently show that attention is a significant moderator of decision weights that relate attribute size to choices in the context of a modified random utility model. Most importantly, we show that a decomposition of the attention data into individual average attention and trial-wise differences in attention to a given attribute adds explanatory power to models of the decision process. Specifically, when considering individual average attention, our findings show that when participants spend more time than the average person looking at an outcome, this increases the importance of this outcome for the decision. In addition, trial-wise deviations in attention also matter: if a subjects spends more time looking at a particular outcome in a given trial (compared to their experiment-wide average focus time on that attribute reflected by the individual average attention), our results indicate that a higher weight is assigned to that outcome during that trial. This result mirrors prior work on the causal effects of attention, showing that manipulating presentation times for a given attribute and on a given trial, leads to a greater influence of that attribute on the decision process (e.g., Hirmas and Engelmann, 2023; Olschewski et al, 2018; Pachur et al, 2018). Although the sample size of our study is comparable with many other studies involving eye-tracking (e.g., Alós-Ferrer and Ritschel, 2022; Devetag et al, 2016; Hausfeld et al, 2021), we nonetheless perform a number of robustness checks. We perform two analyses to explore whether the sample size might affect our results. First, we perform a Monte-Carlo simulation, where we randomly pick sub-samples of different sizes from our total sample and estimate the parameters of our decision model. The results displayed in Appendix section D show that after 60–70 participants or more, the results become stable and stay within the 95% confidence intervals of the full-sample estimations. Moreover, we also inspect whether using only the data from Experiments 1 and 2 generate different results. In Appendix table E, we show that the results remain relatively stable across experimental samples. Specifically, attention remains a significant moderator for the influence of value on choice when using the samples of each experiment, as originally reported for the whole sample.

Since we have established that the attention models produce robust results and have better descriptive power, we next ask whether the attention models also generate better out-of-sample predictions. In order to analyze the predictive power of our models, we perform a simulation exercise in which we randomly select 80% of the trials for each participant, estimate the decision models (baseline and our attention-based models) with that data and predict the decisions of the remaining 20% of the trials. Our results (reported in Appendix F) show that the models including only individual-average attention and the model including both individual and trial-wise attention outperform the baseline model (without attention) in out-of-sample predictions. In addition, we find that individual average attention shows a consistent improvement in predictive power, outperforming the baseline model in 90.5% of the simulations; while the full model, that includes trial-wise deviations only outperforms it in 72.6% of the cases. It is important to note that even though the out-of-sample predictions of the model with individual-average attention are consistently superior, the marginal improvement in the predictions is rather small (from 87.7% correct predictions in the baseline model to 88.0% correct in the individual-average attention model, F3, panel A). We attribute the small differences to the already strong predictive power of the baseline model. When comparing the out-of-sample predictions across participants (F3, panel B), we find that including attention in the model increases the predictive accuracy by up to 2.84 percentage points (individual attention model) and 5.9 percentage points (full model) for those individuals for which the baseline predictions are relatively low. These results show that adding individual average attention to the baseline model leads to modest but consistent improvements in out-of-sample predictions, while the inclusion of trial-wise deviations leads to specific improvements for some of the most volatile participants.

Finally, we would like to point out a number of methodological advances brought forward by our modeling exercise: (1) our rich eye-tracking data gave us an opportunity to compare different attention measures and make inferences on their relative suitability for decision models. We find that a decomposition of the attention data into individual average attention and trial-wise differences in attention leads to superior model fits and modest improvements in predictive power. Our current recommendation for the analyses of attention data obtained via eye-tracking is to use the richer data obtained from the attention channel decomposition outlined here, but future research is required to develop a more nuanced understanding of the different contexts in which each of the attention measures is superior. (2) We compare different modeling approaches from economics and other fields. We find that the inclusion of random slopes can reduce the predictive power of some attention measures as a moderator, but does not eliminate it. In such cases, relative measures, like the proportion of time spent looking at an attribute, are likely the best predictors of individual differences in attribute relevance, consistent with the existing literature (Rahal & Fiedler, 2019). While random slope models are the preferred model type in some fields (e.g., Meteyard & Davies, 2020; Hoven et al, 2023), they increase the complexity of mixed models often to the point of non-convergence (Barr et al., 2013). Our results suggest that in cases when random slopes cannot be modeled—e.g., when the amount of data does not match model complexity—the inclusion of attention variables, specifically individual average attention, may compensate for some of the otherwise unexplained variance.

Among economists, there is some expectation that attention can be a “unifying” variable that ties together hitherto separate phenomena (Gabaix, 2019). Similarly, the potential of attention and eye-tracking are attracting scholars from new research fields, such as management and organization science (e.g., Orquin and Mueller Loose, 2013; Meißner and Oll, 2019), and we expect this trend to increase both for researchers and in commerce as attention measurements such as eye-tracking become cheaper and less challenging to implement, e.g., via advances in online measures of attention via mouselabweb (Willemsen & Johnson, 2011), similar applications in oTree (Hirmas & Engelmann, 2024), and internet-connected webcams (Yang & Krajbich, 2021). Overall, our results support attention as a useful variable to understand the decision making process. We show here that both individual and contextual variation matter, suggesting a role for modeling approaches that emphasize individual agency and salience.

Moreover, the framework we propose here can be flexibly applied to different experimental contexts and can help answer a number of questions that are crucial to fulfill this promise of attention research. For instance, future research should address how the relative influence of contextual versus individual drivers of attention varies across environments? Moreover, how do various aspects of salience affect stimulus-driven attention and the occurrence of behavioral biases? Finally, how do individual differences in attention correlate with personal characteristics and decision parameters? Answering these questions will be valuable to both theorists and policy makers alike. More generally, our approach demonstrates the fruitful interaction between cognitive (neuro-)science and economic analysis.

## Supplementary Information

Below is the link to the electronic supplementary material.

Supplementary file 1 (pdf 1078 KB)

## Footnotes

1 Other recent papers have focused on establishing a causal effect of attention by manipulating attention via visual salience, exposure time or other contextual, bottom-up interventions. Evidence has been presented for such attentional influences on choice in a multitude of domains (see e.g., Armel et al, 2008; Reutskaja et al, 2011; Atalay et al, 2012; Pärnamets et al, 2015; Pachur et al, 2018; Ghaffari and Fiedler, 2018; Gluth et al, 2018; 2020). In economics, Dertwinkel-Kalt et al. (2017) and Dertwinkel-Kalt and Köster (2019) have tested recent models of salience discussed above. These studies have shown that there is a causal effect of attention, although its size is often modest.

2 Nonetheless, there are other measures of attention that can be derived from eye-tracking data. See Rahal and Fiedler (2019), the authors write a comprehensive description of all the attention measures used in the literature and their potential uses.

3 We use the logarithm of the dwell time. This is a common practice when using time variables, or others that are positively skewed (e.g., income).

4 The method is based on the concept of Shapley values from cooperative game theory (Roth, 1988). Its output, the Shapley value, represents the average marginal contribution of a covariate across all possible combinations of covariates based on a defined value function. This analysis is conducted by running regressions with all subsets of covariates and record a value function ($R^2$ in our case to determine percentage of variance explained). Then, we sequentially run separate regressions in which we include one attribute at a time and compute the marginal contribution to the value function. The marginal contribution is calculated for all possible ordering sequences of the covariates, ensuring a fair and consistent method to attribute the amount of variance explained to each covariate.
