# Supplementary material for: Individual and contextual effects of attention in risky choice
Source: Exp Econ. 2024 Sep 30;27(5):1211–38. doi: 10.1007/s10683-024-09849-7 (PMC11897100; doi:10.1007/s10683-024-09849-7)
Supplement: Supplementary file 1 — (pdf 1078 KB) [file 10683_2024_9849_MOESM1_ESM.pdf]

APPENDIX

Individual and Contextual Effects of Attention in Risky Choice

Alejandro Hirmas[1], Jan B. Engelmann[1], Joël van der Weele[1]

[1*]Center for Experimental Economics and political Decision making (CREED), Universiteit van Amsterdam, Amsterdam, The Netherlands.

Appendix A Determinants of attention

Tables A1 and A2 show the estimates of the linear regressions with random effects used to calculate the Shapley values in section 5.1. The different columns show the estimates for the different attention variables, proportion of time (p), log dwell-times (DT), number of fixations (N), first and last fixation.

Table A1: Determinants of attention to Gains

	p(DT)	DT	Last	First	N
trial	-0.0306***	-0.0833***	-0.0770***	-0.00815	-0.0247*
	(-3.29)	(-5.60)	(-5.31)	(-1.25)	(-2.41)
$trial^2$	0.0107	0.0353**	0.0266*	-0.000988	0.00716
	(1.01)	(3.18)	(2.26)	(-0.14)	(0.73)
Gain Value	0.0306***	0.0509***	0.0421**	0.00719	0.0160
	(3.80)	(4.75)	(3.06)	(1.26)	(1.94)
Loss Value	0.0716**	0.0459*	0.0576***	0.0491**	0.0628***
	(3.24)	(2.46)	(3.81)	(2.67)	(3.78)
L. Left	0.393***	0.315***	-0.233***	-1.034***	0.213***
	(5.34)	(6.58)	(-5.11)	(-10.27)	(3.74)
Female	0.0152	0.175	0.0158	0.00523	0.00945
	(0.30)	(1.50)	(0.16)	(0.37)	(0.21)
Age	0.0399**	0.0804*	0.0875*	-0.00724	0.0563***
	(2.97)	(2.04)	(2.44)	(-1.70)	(5.62)
Constant	-0.223***	-0.328**	0.0664	0.521***	-0.131*
	(-3.49)	(-2.99)	(0.70)	(9.69)	(-2.50)
Observations	14238	14238	14238	14238	14238
AIC	39411.4	35914.0	37310.1	35951.0	39938.4
BIC	39487.0	35989.6	37385.8	36026.7	40014.1

t statistics in parentheses
* $p < 0.05$, ** $p < 0.01$, *** $p < 0.001$

Table A2: Determinants of attention to Losses

	p(DT)	DT	Last	First	N
trial	0.0306***	-0.0446**	-0.0570***	0.00815	0.0247*
	(3.29)	(-2.64)	(-3.84)	(1.25)	(2.41)
trial^2	-0.0107	0.0203	0.0233*	0.000988	-0.00716
	(-1.01)	(1.82)	(2.00)	(0.14)	(-0.73)
Gain Value	-0.0306***	0.0132	0.0239	-0.00719	-0.0160
	(-3.80)	(0.96)	(1.71)	(-1.26)	(-1.94)
Loss Value	-0.0716**	-0.0408**	-0.0368**	-0.0491**	-0.0628***
	(-3.24)	(-2.63)	(-2.60)	(-2.67)	(-3.78)
L. Left	-0.393***	-0.181***	0.328***	1.034***	-0.213***
	(-5.34)	(-3.73)	(7.02)	(10.27)	(-3.74)
Female	-0.0152	0.159	-0.00685	-0.00523	-0.00945
	(-0.30)	(1.50)	(-0.07)	(-0.37)	(-0.21)
Age	-0.0399**	0.0304	0.0576*	0.00724	-0.0563***
	(-2.97)	(0.89)	(2.04)	(1.70)	(-5.62)
Constant	0.223***	-0.0450	-0.191*	-0.521***	0.131*
	(3.49)	(-0.49)	(-2.52)	(-9.69)	(2.50)
Observations	14238	14238	14238	14238	14238
AIC	39411.4	36442.7	37307.2	35951.0	39938.4
BIC	39487.0	36518.3	37382.8	36026.7	40014.1

t statistics in parentheses
* $p < 0.05$, ** $p < 0.01$, *** $p < 0.001$

Appendix B Association between attentional loss aversion and individual differences in intercepts

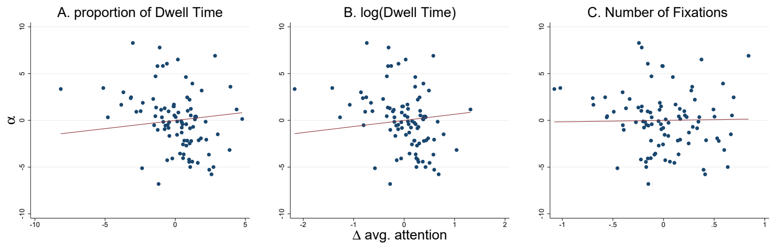

Fig. B1: Avg. Attention and Individual Differences (intercept)

The figures above show the correlation between the differences in decision intercepts (α_i, on the vertical axis) and the differences in average attention towards losses relative to gains ($\bar{a}_{L,i} - \bar{a}_{G,i}$, on the horizontal axis). The red line displays the linear fit between the differences in weights and the differences in attention. The differences in attention are standardized.

Appendix C Testing our Model using different Attention Measures

We run manipulation checks for our results reported in the main text by testing different measures for attention that can be extracted from eye-tracking data. Specifically, tables C3 to C6 show the decision models using as the attention measure the proportion of time, number of fixations, first fixation and last fixation respectively. We largely find overlapping results for the proportion of dwell time and number of fixations, but not for first and last fixation. The measure of individual-average attention shows significant results for the proportion of dwell time (b(L) = 0.070, p-value = 0.001, column

5) and the number of fixations (b(G) = 0.047, p-value = 0.014). The variables proportion of time, first and last fixation are incorporated only for one attribute, since these variables are perfectly colinear (i.e., the model including them is unidentifiable and cannot be estimated). Column (1) is the baseline model without attention (same for all tables). Column (2) uses the attention measure without decomposition, $a(x)$; columns (3) and (4) incorporate the attention measures of individual differences, $\bar{a}(x)$, and trial-wise deviations, $\tilde{a}(x)$, respectively. Column (5) incorporates both measures simultaneously.

Table C3: Decision models including attention measures (Proportion of dwell time)

	(1) Baseline	(2) Attention	(3) Att(ID)	(4) Att(TW)	(5) Att(ID+TW)
Decision					
Gain	0.440***	0.440***	0.440***	0.440***	0.439***
	(0.000)	(0.000)	(0.000)	(0.000)	(0.000)
Loss	0.582***	0.580***	0.579***	0.581***	0.577***
	(0.000)	(0.000)	(0.000)	(0.000)	(0.000)
Loss × $a(L)$		0.005			
		(0.669)			
Loss × $\bar{a}(L)$			0.069***		0.070***
			(0.001)		(0.001)
Loss × $\tilde{a}(L)$				-0.000	0.004
				(0.994)	(0.753)
$a(L)$		-0.089			
		(0.690)			
$\bar{a}(L)$			0.545		0.417
			(0.201)		(0.352)
$\tilde{a}(L)$				-0.177	-0.111
				(0.394)	(0.616)
Constant	-1.588*	-1.623*	-1.564*	-1.643*	-1.597*
	(0.011)	(0.021)	(0.025)	(0.020)	(0.021)
Observations	14238	14238	14238	14238	14238
AIC	8016.990	7986.480	8009.290	7988.911	7979.666
BIC	8100.190	8084.807	8107.618	8087.239	8093.121

p-values in parentheses

* $p < 0.05$, ** $p < 0.01$, *** $p < 0.001$

Table C4: Decision models including attention measures (Number of fixations)

	(1) Baseline	(2) Attention	(3) Att(ID)	(4) Att(TW)	(5) Att(ID+TW)
Decision					
Gain	0.440***	0.441***	0.440***	0.442***	0.442***
	(0.000)	(0.000)	(0.000)	(0.000)	(0.000)
Gain × $a(G)$		-0.005			
		(0.492)			
Gain × $\bar{a}(G)$			0.051**		0.047*
			(0.007)		(0.014)
Gain × $\tilde{a}(G)$				-0.011	-0.008
				(0.123)	(0.244)
Loss	0.582***	0.578***	0.582***	0.578***	0.578***
	(0.000)	(0.000)	(0.000)	(0.000)	(0.000)
Loss × $a(L)$		0.014			
		(0.202)			
Loss × $\bar{a}(L)$			0.031		0.040
			(0.208)		(0.118)
Loss × $\tilde{a}(L)$				0.015	0.015
				(0.179)	(0.179)
$a(G)$		0.262			
		(0.172)			
$\bar{a}(G)$			-0.287		-0.087
			(0.744)		(0.920)
$\tilde{a}(G)$				0.449*	0.367
				(0.025)	(0.063)
$a(L)$		0.180			
		(0.404)			
$\bar{a}(L)$			-0.720		-0.609
			(0.419)		(0.491)
$\tilde{a}(L)$				0.203	0.204
				(0.349)	(0.355)
Constant	-1.588*	-1.695*	-1.628*	-1.746*	-1.762**
	(0.011)	(0.017)	(0.016)	(0.011)	(0.008)
Observations	14238	14238	14238	14238	14238
AIC	8016.990	8003.342	8013.495	7999.017	7997.809
BIC	8100.190	8116.797	8126.950	8112.473	8141.519

p-values in parentheses
* $p < 0.05$, ** $p < 0.01$, *** $p < 0.001$

Table C5: Decision models including attention measures (First Fixation)

	(1) Baseline	(2) Attention	(3) Att(ID)	(4) Att(TW)	(5) Att(ID+TW)
Decision					
Gain	0.440***	0.440***	0.440***	0.440***	0.440***
	(0.000)	(0.000)	(0.000)	(0.000)	(0.000)
Loss	0.582***	0.582***	0.583***	0.582***	0.583***
	(0.000)	(0.000)	(0.000)	(0.000)	(0.000)
Loss × $a(L)$		-0.011			
		(0.363)			
Loss × $\bar{a}(L)$			-0.026		-0.037
			(0.396)		(0.247)
Loss × $\tilde{a}(L)$				-0.009	-0.011
				(0.463)	(0.370)
$a(L)$		-0.270			
		(0.259)			
$\bar{a}(L)$			0.026		-0.240
			(0.962)		(0.678)
$\tilde{a}(L)$				-0.222	-0.267
				(0.321)	(0.263)
Constant	-1.588*	-1.608*	-1.716*	-1.632*	-1.736*
	(0.011)	(0.024)	(0.013)	(0.021)	(0.013)
Observations	14238	14238	14238	14238	14238
AIC	8016.990	8015.414	8016.231	8015.574	8014.661
BIC	8100.190	8113.741	8114.559	8113.902	8128.116

p-values in parentheses

* $p < 0.05$, ** $p < 0.01$, *** $p < 0.001$

Table C6: Decision models including attention measures (Last Fixation)

	(1) Baseline	(2) Attention	(3) Att(ID)	(4) Att(TW)	(5) Att(ID+TW)
Decision					
Gain	0.440***	0.439***	0.440***	0.439***	0.439***
	(0.000)	(0.000)	(0.000)	(0.000)	(0.000)
Loss	0.582***	0.581***	0.582***	0.581***	0.580***
	(0.000)	(0.000)	(0.000)	(0.000)	(0.000)
Loss × $a(L)$		-0.010			
		(0.201)			
Loss × $\bar{a}(L)$			0.027		0.020
			(0.216)		(0.402)
Loss × $\tilde{a}(L)$				-0.010	-0.010
				(0.181)	(0.195)
$a(L)$		-0.292			
		(0.053)			
$\bar{a}(L)$			-0.167		-0.396
			(0.687)		(0.354)
$\tilde{a}(L)$				-0.286	-0.294
				(0.050)	(0.052)
Constant	-1.588*	-1.593*	-1.647*	-1.583*	-1.649*
	(0.011)	(0.024)	(0.017)	(0.027)	(0.017)
Observations	14238	14238	14238	14238	14238
AIC	8016.990	8008.012	8016.114	8009.204	8007.300
BIC	8100.190	8106.340	8114.442	8107.531	8120.756

p-values in parentheses
* $p < 0.05$, ** $p < 0.01$, *** $p < 0.001$

Appendix D Sample size and parameters' stability

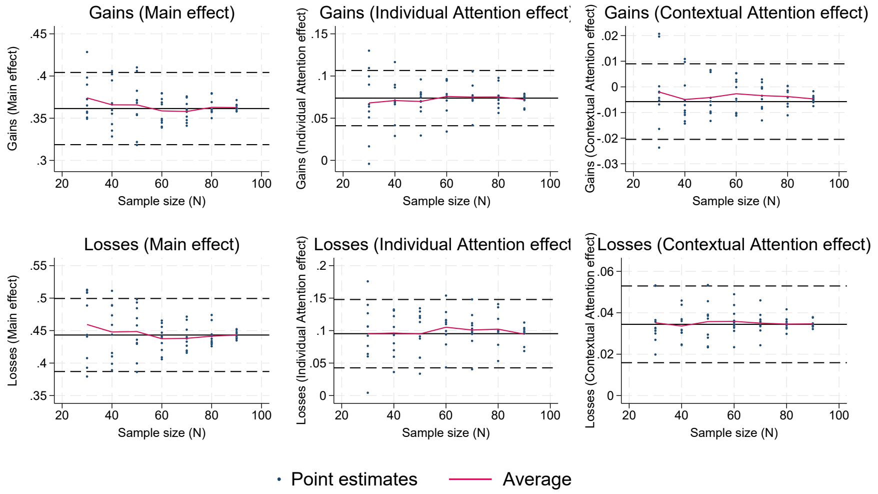

Fig. D2: Estimations of parameters by sample size

In Figure D2, we present 1,000 simulations, where we randomly select N participants without replacement, with $N \in \{30, 40, 50, 60, 70, 80, 90\}$ and estimate the model described by equation (5). The panels in the first row display the parameters for the gain values, and the second row for the loss values. The first column shows the the main effects (ω_G and ω_L). The second and third columns show the effects of individual-average attention ($\pi_{G,\bar{a}}$ and $\pi_{L,\bar{a}}$) and trial-wise deviations ($\pi_{G,\tilde{a}}$ and $\pi_{L,\tilde{a}}$) respectively.

Appendix E Estimations by experiment

Table E7 shows the estimations of our full decision model using the data from each experiment separately. The first column presents the full model using the whole sample, while column two and three use the data from experiment 1 and 2 respectively. We use dwell-times as the attention variable to stay consistent with the results reported in tables throughout the main text.

Table E7: Estimation of decision model for each experiment

	(1)<br>Full Sample	(2)<br>Experiment 1	(3)<br>Experiment 2
Decision			
Gain	0.361***	0.370***	0.357***
	(0.000)	(0.000)	(0.000)
Gain × $\bar{a}(G)$	0.074***	0.078**	0.067***
	(0.000)	(0.007)	(0.000)
Gain × $\tilde{a}(G)$	-0.006	-0.007	-0.006
	(0.448)	(0.585)	(0.546)
Loss	0.443***	0.463***	0.439***
	(0.000)	(0.000)	(0.000)
Loss × $\bar{a}(L)$	0.095***	0.129***	0.062*
	(0.000)	(0.001)	(0.022)
Loss × $\tilde{a}(L)$	0.034***	0.049**	0.022*
	(0.000)	(0.002)	(0.038)
$\bar{a}(G)$	-0.362	-0.385	-0.123
	(0.545)	(0.714)	(0.842)
$\tilde{a}(G)$	0.337	0.334	0.361
	(0.132)	(0.332)	(0.221)
$\bar{a}(L)$	-0.052	0.426	-0.556
	(0.941)	(0.711)	(0.435)
$\tilde{a}(L)$	0.519**	0.792*	0.301
	(0.003)	(0.010)	(0.137)
Constant	-2.593***	-2.219**	-2.784***
	(0.000)	(0.002)	(0.000)
Observations	14238	6169	8069
AIC	8575.132	3817.773	4763.299
BIC	8665.896	3898.501	4847.249

p-values in parentheses
* $p < 0.05$, ** $p < 0.01$, *** $p < 0.001$

Appendix F Out-of-sample predictions

In order to assess the predictive power of our random intercept attention models (see Table 5), we perform the following Monte-Carlo simulation. In 1000 simulations, we randomly select 80% of the trials (without replacement) to estimate the decision models. Specifically, we estimate the baseline model with no attention measures, the model that incorporates individual attention, and the model with both attention measures, individual and trial-wise attention. Then, we use these models to predict the choice of participants in the remaining 20% of the observations. Figure F3 shows the model comparisons across all simulations. In Panel A, we can see the distribution of average correct out-of-sample predictions by each model. The model including the individual-average attention index is the best performing model on average (88% correct), followed by the model including both individual and trial-wise attention (87.9% correct), compared to the baseline model (87.7% correct).

It is important to note that even the baseline model already can make very strong out-of-sample predictions, making it more difficult to marginally improve these predictions. Nonetheless, in 905 from the 1000 simulations, the predictions of the model including individual average attention outperformed the baseline model. The full model with both indexes of attention outperforms the baseline model only in 726 out of 1000. These results suggest that the individual average attention effects have a robust predictive power on the decisions, while trial-wise deviations of attention, moderately predict the choice.

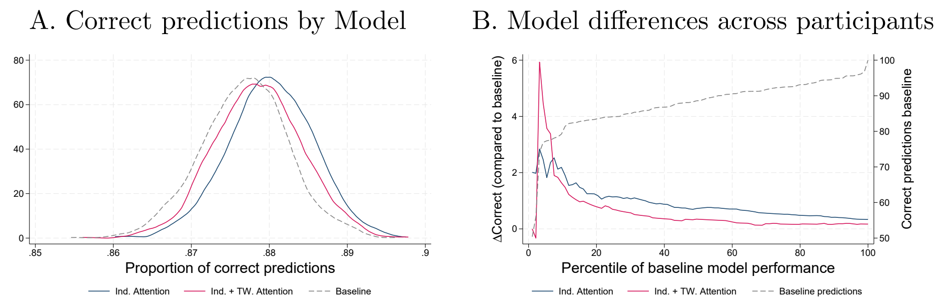

Fig. F3: Out-of-Sample predictive accuracy

Panel B shows the predictive performance of the attention models relative to the baseline model. More specifically, the horizontal axis shows participant numbers (in percentiles) that were sorted based on the predictive performance of the baseline model fitted to their individual data. In the context of this plot, this value is equivalent to the percentile of the baseline model's predictive performance, with increasing baseline model performance from left to right. The left vertical axis shows the difference in the percentage of correct predictions between baseline and attention models based on a running mean of this difference (delta correct compared to baseline) at a given percentile of the baseline model fit performance (reflected on the x-axis). The right vertical axis shows the percentage of correct predictions of the baseline model for a given percentile. The improvements in predictive power by the attention models are rather modest for the overall sample (overall average improvement = 0.3 percentage points for the individual attention model shown in red), but can go up to 2.84 percentage points for the individual attention model (blue line) and 5.9 percentage points for the full model (red line) for subsamples that show more modest baseline model performance. These results support the idea that attention can lead to larger improvements in predictive power for individuals with more inconsistent choices or when the decisions of the individuals are more heterogeneous (and therefore the baseline model predictions are lower).

Appendix G Supplementary Figures

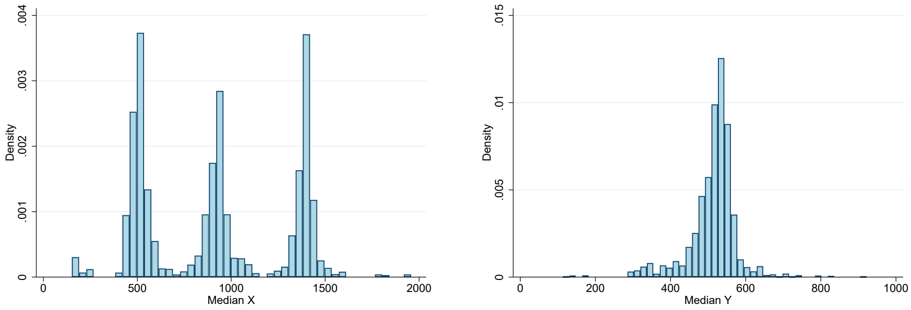

Fig. G4: Horizontal and Vertical Clusters of visual fixations

The figures above describe the center of the individual clusters on the x- and y-axis of the screen. Left Panel: three main clusters for the horizontal axis, consistent with the regions of interest (left, middle and right). Right panel: on the vertical axis, there is only one concentration point since all regions of interest are aligned at the same height.

Information Brochure for Decision-Making Study

Dear participant ,

Thank you for participating in this experiment. Before you start the experiment, it is important that you are aware of the procedures followed in this study. Please read the following text carefully and do not hesitate to ask your experimenter if you have any questions.

Aim of the study
The goal of our experiment is to investigate how people make financial decisions under risk. The experiment will take about 1 hour to complete and we will track your eye movements throughout the experiment.

Experiment procedure
You will receive an initial payment of 10 Euros for filling out a number of questionnaires. Based on your decisions throughout the experiment, you have the chance to earn additional money, as well as to lose money from your endowment. This is because one trial will be randomly selected at the end of the experiment - the payout relevant trial. The decision you made on this trial will be realized as explained in detail below. All values shown in the experiment are in *monetary units* (MU), which have an exchange rate of 1 MU = 0.1851852 Euros.

You are not allowed to write anything down or make notes during the experiment. Moreover, it is very important that you look at the screen throughout the experiment, unless there is a break and we ask you to relax your eyes.

a. Detailed description of the choice scenarios

The experiment consists of a total of 160 decisions. Your task is to make a decision about which of two options you prefer: (A) receiving a certain payout, which leads to no change to your endowment of 10 Euros, or (B) playing a lottery, which can lead to additional earnings with a 50% probability, but also losses with a 50% probability. Choosing the lottery means that you could win, or lose, one of the amounts displayed on the screen with equal (50%) probability. Note that the values offered by the lottery will change on every trial, so please make sure that you pay attention to the amounts on every trial before you make a decision. The certain payout, on the other hand, will remain the same throughout the experiment, such that when you choose this option there will be no additional earnings added to or losses subtracted from your initial endowment.

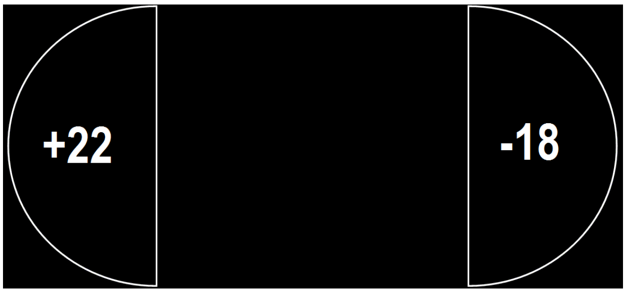

To make this even clearer, consider the following example: On every trial, the values of the lottery will be displayed on the screen as shown in the figure on the right. In this example trial, gains are shown on the left side (gains are signified by “+”) and losses are on the right (losses are signified by “-“). This means that if you decide to accept the lottery on this trial, you will have a 50% chance of winning 22 MU and a 50% chance of losing 18 MU, which will be added to or subtracted from your

endowment (your payment of 10€ for the questionnaires). Whether you receive the gain or loss will be decided upon via a virtual coin flip, if you selected the lottery on the payout relevant trial selected at the end of the experiment. Note that the locations of gains and losses are not set and can also be reversed on some trials, with losses on the left and gains on the right. Choosing the safe option always leads to no change from your initial endowment, that means you do not receive any additional gains, nor will you incur any additional losses.

Once you have decided which option you prefer, you can communicate your choice by pressing one of two buttons:

- Press the **up arrow key** to choose the lottery.
- Press the **down arrow key** to choose the safe option.

You will receive a brief feedback after you made the decision (for ca. 1 second), which indicates what option you have chosen, such that the letter **L** appears in the center of the screen, when you chose the lottery, and the letter **C** appears in the center of the screen when you chose the certain payout. After a short break, the next lottery will be displayed.

b. Details on payout determination

After you have made your choice for all 160 lotteries, you will select the payout relevant trial by rolling three 10-sided dice. The die rolls will reflect a number between 1 and 160 (the number of all decisions that you have made) as follows: the first die that you roll indicates whether your payout relevant trial is smaller than 100 (die shows a number <5), or greater than 100 (die shows a number >= 5). The second and third die rolls then determine the exact trial number. If the trial number is greater than 160, you will roll dice 2 and 3 until a number =< 160 is generated. You will then enter the chosen trial number into the computer, which will recall the exact decision that you have made on that trial.

If you chose to play the lottery, a computer algorithm equivalent to an even coin flip will determine whether the gain amount on this trial will be added to your endowment, or whether the loss amount will be deducted from your endowment. Please remember that the monetary units will first be converted to euros using the exchange rate of 1 MU = 0.1851852 Euros. If you chose the certain option, you will receive your endowment of 10 Euros. The amounts on the randomly selected payout relevant trial, your decision and your additional wins or losses will be displayed to you on the screen. Your final payment will be calculated as follows:

If the outcome was a gain: 10 Euro (endowment) + gain amount * 0.1851852
If the outcome was a loss: 10 Euro (endowment) - loss amount * 0.1851852
If you chose the certain outcome: 10 Euro (endowment).

c. Subparts of the experiment

1. At the beginning of the experiment you will fill out questionnaires for about 30 minutes. For your work, you will receive a payment of 10 Euros for use in the following part of the experiment.
2. After the questionnaires, you will be given the chance to familiarize yourself with the experiment in 10 practice trials. These 10 decisions will not affect your final payout and will be made solely for the purpose of giving you experience with the choice scenarios and the speed of the experiment.
3. The main experiment begins after all your questions have been answered and we are certain that you have understood all aspects of the experiment. We will now set up the eye tracker, which monitors where you are looking throughout the remainder of the experiment. To this end, we will ask you to place your head on a chin rest. From this point on, it is very important that you move your head as little as possible and fixate on the screen.
4. At the end of the experiment, we ask you to fill out a final questionnaire, which will take an additional 10 minutes.
5. Finally, you will receive your payment, which will be determined as outlined in detail above.

Confidentiality

All research data will remain completely confidential. In case of either using these results in scientific publications or making these results public in any other way, this will happen anonymously. Personal data will not be seen by others without explicit approval.

VOLUNTARY

Your participation in this study is voluntary. You are free to choose whether to participate in this study. You may also choose to withdraw from the study or to decline to answer any questions at any time. You will not be penalized or lose any benefits to which you are otherwise entitled if you choose not to participate or choose to withdraw.

INSURANCE

Participation in this study involves making simple choices which is routinely used and will do no harm to your health or safety. Because this study poses no risks to your health or safety, the conditions of the regular liability insurance of the University of Amsterdam are applied.

FURTHER INFORMATION

If you have questions about this research beforehand or afterwards, please contact the responsible researcher dr. Jan Engelmann (e-mail j.b.engelmann@gmail.com). In case of complaints about this study, you can contact Dr. Wery van den Wildenberg, member of the ethical committee of the Psychology Department of the University of Amsterdam (Fmg-UvA, REC-G1.10, Nieuwe Achtergracht 129 B, 1018 WS Amsterdam, 020-5256686, w.p.m.vandenwildenberg@uva.nl).

AGREEMENT

When you sign this document containing a written explanation of the experiment that you are participating in, you declare that you have read and understood the instructions and that all your questions have been answered by the experimenter. Moreover, with your signature you agree to participate in the procedures outlined in the instruction above.

If you have further questions about this experiment, please contact the responsible researcher dr. Jan Engelmann (e-mail j.b.engelmann@gmail.com). In case of complaints about this study, you can contact Dr. Wery van den Wildenberg, member of the ethical committee of the Psychology Department of the University of Amsterdam (Fmg-UvA, REC-G1.10, Nieuwe Achtergracht 129 B, 1018 WS Amsterdam, 020-5256686, w.p.m.vandenwildenberg@uva.nl).

[**Participant**]

"I have read and understood the information above and agree to participate in the current experiment and grant the experimenters permission to use my data. I reserve the right to withdraw from this agreement without giving any explanation, as well as to withdraw from participation in this experiment at any time."

Date:

…………………………….......... ……………………………

Participant name *Signature*

[**Experimenter**]

"*I have explained the experiment to the participant. I will answer any further questions to my best knowledge.*"

Date:

…………………………….......... ……………………………

Researcher name *Signature*

Exit Questionnaire.

Thank you again for participating in our experiment. Because we are always concerned with improving the experiment and the instructions, we have just a few questions for you. Please rate how you much you agree with the following statements using the scale below.

**0**	**1**	**2**	**3**	**4**
Strongly disagree	Disagree	Undecided	Agree	Strongly agree

**Statement**	**Your Evaluation**
During the experiment, I *never considered* that I would not receive the amount that I selected via dice rolls at the end the end of the experiment.	
During the experiment, I *considered* that the experiment was programed in such a way that would make me lose money.	
During the experiment, I *never thought* that I was being deceived by the experimenters about the additional money I could win or lose?	
During the experiment, I *fully understood* that the values shown would be converted to Euros via an exchange rate.	

- Have you ever participated in an experiment in which you were deceived? Please circle your answer.

  Yes No Cannot tell / do not remember

- To what extent do you think that previous experiences with deception influenced your behavior in the current experiment? Please circle one answer. Previous experiences with deception influenced me in this experiment …

**0**	**1**	**2**	**3**	**4**
Not at all	Slightly	Somewhat	Moderately	Extremely

- Please use the space below if you have any other comments or questions about the experiment.

References

Hirmas A, Engelmann J (2024) Learning the value of Eco-Labels: The role of information in sustainable decisions. Available at SSRN 4788773
